# Prognosis of Gleason score 8 prostatic adenocarcinoma in needle biopsies: a nationwide population-based study

**DOI:** 10.1007/s00428-024-03810-y

**Published:** 2024-04-29

**Authors:** Lars Egevad, Chiara Micoli, Brett Delahunt, Hemamali Samaratunga, Andri Wilberg Orrason, Hans Garmo, Pär Stattin, Martin Eklund

**Affiliations:** 1 Department of Oncology-Pathology, Karolinska Institutet, Karolinska University Hospital, 171 76 Stockholm, Sweden; 2 Department of Medical Epidemiology and Biostatistics, Karolinska Institutet, Stockholm, Sweden; 3 Malaghan Institute of Medical Research, Wellington, New Zealand; 4 Aquesta Uropathology and University of Queensland, Brisbane, QLD Australia; 5 Department of Surgical Sciences, Uppsala University, Uppsala, Sweden; 6 Regional Cancer Centre Mid-Sweden, Uppsala University Hospital, Uppsala, Sweden

**Keywords:** Gleason grade, Mortality, Needle biopsy, Prostate cancer

## Abstract

A 5-tier grouping of Gleason scores has recently been proposed. Studies have indicated prognostic heterogeneity within these groups. We assessed prostate cancer-specific mortality (PCSM) and all-cause mortality (ACM) for men diagnosed with Gleason score 3 + 5 = 8, 4 + 4 = 8 and 5 + 3 = 8 acinar adenocarcinoma on needle biopsy in a population-based national cohort. The Prostate Cancer data Base Sweden 5.0 was used for survival analysis with PCSM and ACM at 5 and 10 years as endpoints. Multivariable Cox regression models controlling for socioeconomic factors, stage and primary treatment type were used for PCSM and ACM. Among 199,620 men reported with prostate cancer in 2000–2020, 172,112 were diagnosed on needle biopsy. In 18,281 (11%), there was a Gleason score of 8 in needle biopsies, including a Gleason score of 3 + 5, 4 + 4 and 5 + 3 in 11%, 86% and 2.3%, respectively. The primary treatment was androgen deprivation therapy (55%), deferred treatment (8%), radical prostatectomy (16%) or radical radiotherapy (21%). PCSM in men with Gleason scores of 3 + 5, 4 + 4 and 5 + 3 at 5 years of follow-up was 0.10 (95% *CI* 0.09–0.12), 0.22 (0.22–0.23) and 0.32 (0.27–0.36), respectively, and at 10 years 0.19 (0.17–0.22), 0.34 (0.33–0.35) and 0.44 (0.39–0.49), respectively. There was a significantly higher PCSM after 5 and 10 years in men with Gleason score 5 + 3 cancers than in those with 4 + 4 and in Gleason score 4 + 4 cancers than in those with 3 + 5. Grouping of Gleason scores will eliminate the prognostic granularity of Gleason scoring, thus diminishing the prognostic significance of this proposed grading system.

**Supplementary Information:**

The online version contains supplementary material available at 10.1007/s00428-024-03810-y.

## Background

Prostate adenocarcinoma is often morphologically heterogenous, consisting of two or more grades or patterns as defined in the Gleason grading system and its various amendments. Gleason scoring, which is the sum of the primary and secondary grades, was specifically designed to take the various grades present into account in order to provide an overall prognostic assessment of individual cases and requires assessment of the whole tumour. It is an important strength of Gleason grading that it factors in the heterogeneity of the tumour. While combinations of Gleason grade do provide an overview of the morphology of the tumour, the Gleason sum score is less granular on its own, and it is evident that identical individual Gleason scores may be achieved through the presence of apparently unrelated grade combinations. This issue is especially evident in cancers showing morphology of the higher grades. While Gleason scoring has the effect of masking morphological complexity, quotation of the composite grades in conjunction with the score, e.g. 3 + 4 = 7, does provide data that are of clinical utility when reporting a tumour as Gleason score 7. Such information is less evident when tumours are reported according to the five recently described International Society of Urological Pathology (ISUP) grades (also known as grade groups). This grading system was specifically introduced to assist patients understand that Gleason score 6 tumour is now the lowest grade, being designated as ISUP grade 1. This grading classification does differentiate between Gleason score 3 + 4 = 7 and 4 + 3 = 7 (ISUP grades 2 and 3, respectively), but there is no such distinction possible for tumours of higher score [1]. As a consequence, a tumour designated ISUP grade 4 may in reality be Gleason score 3 + 5, 4 + 4 or 5 + 3, each of which possibly reflect a differing prognosis. In the past, several studies have indicated a potential prognostic heterogeneity among these grade combinations [2–8], but there has been considerable disagreement as to whether there is a similar outcome for some specific combinations, e.g. 3 + 5 and 4 + 4 [6], 4 + 4 and 5 + 3 [2] or 3 + 5 and 5 + 3 [3, 4].

The aim of this study was to investigate prostate cancer-specific and all-cause mortality among subgroups of Gleason score 8 by analysing Gleason scores 3 + 5, 4 + 4 and 5 + 3 in a large population-based cohort taking date of diagnosis and treatment into account.

## Materials and methods

The National Prostate Cancer Register (NPCR) in Sweden holds clinical and histopathological data on all prostate cancers diagnosed since 1998 with the aim to increase quality of care and adherence to guidelines [9, 10]. The coverage has been estimated to be more than 96% of all prostate cancers diagnosed in Sweden during this period [11]. For this study, men diagnosed with acinar adenocarcinoma, which were reported to NPCR between the inclusive years 2000–2020, were identified. From this group, all cases of acinar adenocarcinoma with Gleason scores 8 (4 + 4, 3 + 5 or 5 + 3) in needle biopsies were included in the study. Data were extracted utilising a similar methodology as in our recent registry study on cancers with Gleason scores 9 to 10 [12]. Briefly, 18,988 cases (9.5%) with incomplete or inconsistent data entries were excluded from further analysis. In addition to Gleason grade/scores, NPCR was searched for data relating to patient age, clinical TNM categories and serum prostate-specific antigen (PSA) levels at presentation. The primary treatment was classified as androgen deprivation therapy (ADT), deferred treatment (DT), radical prostatectomy (RP) or radical radiotherapy (RRT).

In Prostate Cancer data Base Sweden (PCBaSe), 5.0 data in NPCR has been linked to other nationwide population-based health care registers and demographic databases such as the cause of death register, the patient register and the Longitudinal Integrated Database for Health Insurance and Labour Market Studies (LISA), a socioeconomic database, by using the Swedish personal identity numbers as identifiers [10]. Deaths were recorded as death due to prostate cancer or death due to other causes. In brief, comorbidity was assessed by use of the Charlson co-morbidity index (CCI) based on data retrieved from the patient register for the 10-year period before the diagnosis of prostate cancer, as previously described [13, 14].

Data on education level were extracted from the Longitudinal Integrated Database for Health Insurance and Labour Market Studies (LISA) of Statistics Sweden [15].

This study was approved by Research Ethics Authority.

### Statistical analysis

Multivariable Cox regression models were used for time-to-event analyses of death from prostate cancer and death from all causes and were censored at 10 years of follow-up. Multivariable analyses included Gleason score; patient age; T, N and M clinical staging categories; Charlson co-morbidity index; primary treatment; period of diagnosis; and education level.

Prostate cancer-specific mortality (PCSM) and all-cause mortality (ACM) were calculated at 5 and 10 years, using cumulative incidence functions (CIF) that considered competing events (death from other causes than prostate cancer) for PCSM using the nonparametric Aalen-Johansen estimator. Time interval from diagnosis to death was registered. The last death record was from March 2023. Follow-up was calculated from date of diagnosis to date of death, date of emigration or last date of follow-up 2023–04-01 whichever came first. Graphs were generated for PCSM and ACM.

## Results

During the period of the study, a total of 199,620 men were reported to the NPCR with newly diagnosed prostate cancer. Following exclusion of cases where there was incomplete data entry or where cancers were diagnosed in transurethral resection specimens (Suppl. Figure 1), 172,112 men with prostate cancer diagnosed on needle biopsy remained for analysis. Of these, 18,281 (11%) were Gleason score 8. This group of cases consisted of 2085 (11%) Gleason score 3 + 5, 15,776 (86%) Gleason score 4 + 4 and 420 (2.3%) of Gleason score 5 + 3 tumours. The mean age at diagnosis was 71.5, 73.3 and 73.3 years in men with Gleason score tumours of 3 + 5, 4 + 4 and 5 + 3, respectively, while the median PSA values were 12, 23 and 23 ng/ml in men with Gleason scores of 3 + 5, 4 + 4 and 5 + 3, respectively. The distribution of T, N and M stage was quite similar between the three subgroups. When all of the cases in the series were grouped according to the year of diagnosis, the proportion of cancers that were assigned a Gleason score of 8 was 11% (4068/35,665) in 2000–2005, 11% (8745/78,112) in 2006–2014 and 9% (5468/58,335) in 2015–2020 (Suppl. Figure 2). Other clinical and demographic data are shown in Table 1. For men with Gleason score 8 tumours, the most common primary treatment was androgen deprivation therapy (55%), while 21% underwent radical radiotherapy and 16% radical prostatectomy. Deferred treatment was used as primary treatment strategy in 8% of cases.

**Table 1:** Descriptive data of men diagnosed with prostate cancer Gleason score 3 + 5, 4 + 4 and 5 + 3 in Prostate Cancer data Base Sweden (PCBaSe) 4.0 in 2000–2020

		All GS 8	GS 3 + 5	GS 4 + 4	GS 5 + 3
		*n* (%)	*n* (%)	*n* (%)	*n* (%)
Total		18,281 (100.0)	2085 (11.4)	15,776 (86.3)	420 (2.3)
Age, years	< = 70	7101 (38.8)	953 (45.7)	5988 (38.0)	160 (38.1)
	71–80	3999 (21.9)	317 (15.2)	3587 (22.7)	95 (22.6)
	> 80	7181 (39.3)	815 (39.1)	6201 (39.3)	165 (39.3)
Education	Low (elementary school)	7539 (41.2)	744 (35.7)	6626 (42.0)	169 (40.2)
	Intermediate (high school)	6872 (37.6)	840 (40.3)	5861 (37.2)	171 (40.7)
	High (university)	3870 (21.2)	501 (24.0)	3289 (20.8)	80 (19.0)
Civil status	Married	11,593 (64.2)	1295 (62.6)	10,035 (64.4)	263 (64.5)
	Unmarried	2001 (11.1)	279 (13.5)	1676 (10.8)	46 (11.3)
	Divorced	2451 (13.6)	305 (14.7)	2095 (13.4)	51 (12.5)
	Widower	2019 (11.2)	191 ( 9.2)	1780 (11.4)	48 (11.8)
Income	Q1	5577 (31.6)	582 (28.6)	4864 (32.0)	131 (33.2)
	Q2	5096 (28.9)	569 (28.0)	4416 (29.0)	111 (28.2)
	Q3	3799 (21.5)	489 (24.1)	3230 (21.2)	80 (20.3)
	Q4	3175 (18.0)	392 (19.3)	2711 (17.8)	72 (18.3)
Year of diagnosis	2000–2005	4068 (22.3)	240 (11.5)	3688 (23.4)	140 (33.3)
	2006–2014	8745 (47.8)	756 (36.3)	756 (36.3)	756 (36.3)
	2015–2020	5468 (29.9)	1089 (52.2)	1089 (52.2)	1089 (52.2)
S-PSA (ng/ml)	0–3	264 (1.5)	32 (1.5)	221 (1.4)	11 (2.6)
	3.1–10	4903 (27.1)	880 (42.6)	3928 (25.2)	95 (22.8)
	10.1–20	3675 (20.3)	450 (21.8)	3134 (20.1)	91 (21.9)
	> 20	9252 (51.1)	705 (34.1)	8328 (53.3)	219 (52.6)
T category	T1c	4234 (23.2)	592 (28.4)	3566 (22.6)	76 (18.1)
	T2	6309 (34.5)	925 (44.4)	5243 (33.2)	141 (33.6)
	T3	6140 (33.6)	464 (22.3)	5515 (35.0)	161 (38.3)
	T4	1285 (7.0)	63 (3.0)	1189 (7.5)	33 (7.9)
	TX	313 (1.7)	41 (2.0)	263 (1.7)	9 (2.1)
N category	N0	5174 (28.3)	935 (44.8)	4135 (26.2)	104 (24.8)
	N1	1350 (7.4)	151 (7.2)	1153 (7.3)	46 (11.0)
	NX	11,757 (64.3)	999 (47.9)	10,488 (66.5)	270 (64.3)
M category	M0	10,590 (57.9)	1618 (77.6)	8758 (55.5)	214 (51.0)
	M1	3489 (19.1)	221 (10.6)	3172 (20.1)	96 (22.9)
	MX	4202 (23.0)	246 (11.8)	3846 (24.4)	110 (26.2)
Charlson co-morbidity index	0	12,832 (70.2)	1503 (72.1)	11,025 (69.9)	304 (72.4)
	1	3619 (19.8)	386 (18.5)	3154 (20.0)	79 (18.8)
	2 +	1830 (10.0)	196 (9.4)	1597 (10.1)	37 (8.8)
Treatment	Androgen deprivation therapy	10,048 (55.0)	761 (36.5)	9024 (57.2)	263 (62.6)
	Deferred treatment	1413 (7.7)	134 ( 6.4)	1255 ( 8.0)	24 (5.7)
	Radical prostatectomy	2913 (15.9)	491 (23.5)	2373 (15.0)	49 (11.7)
	Radical radiotherapy	3907 (21.4)	699 (33.5)	3124 (19.8)	84 (20.0)
Death status	Alive	6940 (38.0)	1245 (59.7)	5580 (35.4)	115 (27.4)
	Death from prostate cancer	5894 (32.2)	334 (16.0)	5372 (34.1)	188 (44.8)
	Death from other causes	5447 (29.8)	506 (24.3)	4824 (30.6)	117 (27.9)

**Fig. 1:**
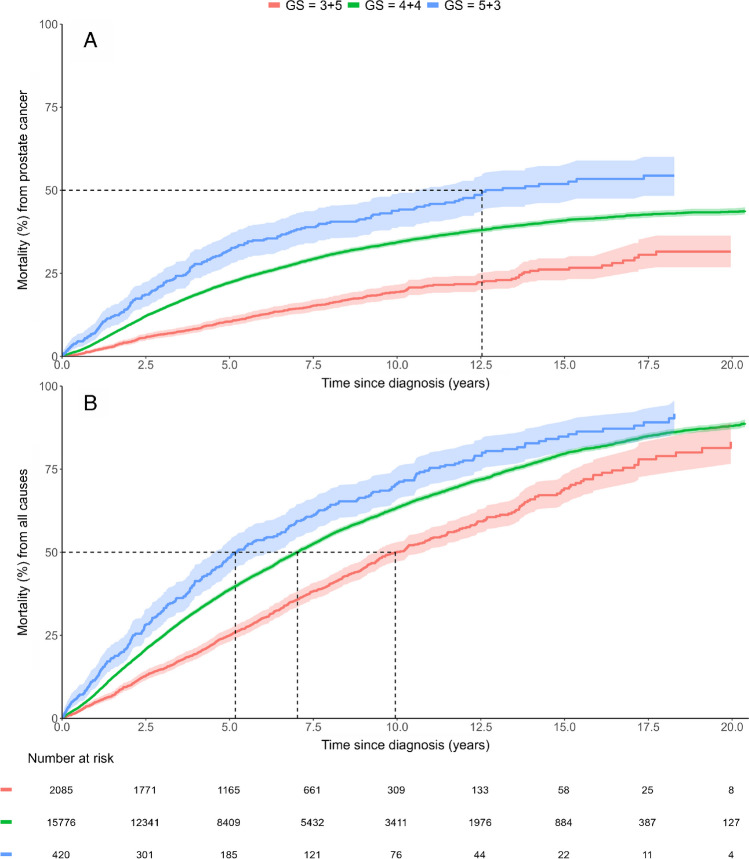
**A** Prostate cancer-specific mortality (PCSM) of men with Gleason score (GS) 3 + 5, 4 + 4 and 5 + 3. **B** All-cause mortality (ACM) of men with Gleason score 3 + 5, 4 + 4 and 5 + 3. Curves are truncated at the time point of the last event

Survival data were available for all 18,281 men with Gleason score 8 tumour, and 38% of men were alive after a median follow interval of 10.8 years (*Q1* = 6.6, *Q3* = 15.0). During this same period, 32% men died of prostate cancer, and 30% died of other causes. The PCSM and ACM after 5- and 10-year follow-up are shown in Table 2. PCSM for men with Gleason scores 3 + 5, 4 + 4 and 5 + 3 at 5 years of follow-up were 0.10 (95% *CI* 0.09–0.12), 0.22 (0.22–0.23) and 0.32 (0.27–0.36), respectively, and at 10 years of follow-up 0.19 (95% *CI* 0.17–0.22), 0.34 (0.33–0.35) and 0.44 (0.39–0.49), respectively. ACM for men with Gleason 3 + 5, 4 + 4 and 5 + 3 at 10 years of follow-up was 0.50 (95% *CI* 0.47–0.53), 0.63 (0.63–0.64) and 0.70 (0.65–0.75), respectively (Fig. 1).

**Table 2:** Prostate cancer-specific mortality (PCSM) and all-cause mortality (ACM) at 5 and 10 years

		5-year mortality							
		Prostate cancer-specific mortality				All-cause mortality			
		All GS 8	GS 3 + 5	GS 4 + 4	GS 5 + 3	All GS 8	GS 3 + 5	GS 4 + 4	GS 5 + 3
	Total	0.21 (0.21–0.22)	0.10 (0.09–0.12)	0.22 (0.22–0.23)	0.32 (0.27–0.36)	0.37 (0.37–0.38)	0.25 (0.23–0.27)	0.39 (0.38–0.40)	0.48 (0.43–0.53)
Treatment	ADT	0.34 (0.33–0.35)	0.25 (0.22–0.28)	0.34 (0.33–0.35)	0.45 (0.39–0.51)	0.56 (0.55–0.57)	0.49 (0.45–0.52)	0.56 (0.55–0.57)	0.65 (0.59–0.70)
	DT	0.14 (0.12–0.16)	0.08 (0.04–0.14)	0.14 (0.13–0.16)	0.26 (0.11–0.45)	0.40 (0.37–0.43)	0.33 (0.24–0.41)	0.40 (0.37–0.43)	0.72 (0.49–0.86)
	RP	0.03 (0.02–0.03)	0.01 (0.00–0.02)	0.03 (0.02–0.04)	0	0.07 (0.06–0.08)	0.06 (0.04–0.09)	0.07 (0.06–0.08)	0.04 (0.01–0.12)
	RRT	0.04 (0.03–0.04)	0.01 (0.01–0.02)	0.04 (0.04–0.05)	0.07 (0.03–0.14)	0.11 (0.10–0.12)	0.09 (0.07–0.12)	0.11 (0.10–0.12)	0.12 (0.06–0.21)
Year of diagnosis	2000–2005	0.28 (0.27–0.29)	0.23 (0.18–0.29)	0.28 (0.26–0.29)	0.43 (0.35–0.51)	0.47 (0.45–0.48)	0.46 (0.40–0.52)	0.46 (0.45–0.48)	0.57 (0.49–0.65)
	2006–2014	0.22 (0.21–0.23)	0.10 (0.08–0.12)	0.23 (0.22–0.24)	0.29 (0.23–0.36)	0.38 (0.37–0.39)	0.24 (0.21–0.27)	0.4 (0.39–0.41)	0.48 (0.40–0.55)
	2015–2020	0.14 (0.13–0.15)	0.07 (0.06–0.09)	0.16 (0.14–0.17)	0.20 (0.13–0.29)	0.28 (0.27–0.29)	0.2 (0.18–0.23)	0.3 (0.28–0.31)	0.35 (0.25–0.45)
		10-year mortality							
		Prostate cancer-specific mortality				All-cause mortality			
		All GS 8	GS 3 + 5	GS 4 + 4	GS 5 + 3	All GS 8	GS 3 + 5	GS 4 + 4	GS 5 + 3
	Total	0.33 (0.32–0.34)	0.19 (0.17–0.22)	0.34 (0.33–0.35)	0.44 (0.39–0.49)	0.62 (0.61–0.63)	0.50 (0.47–0.53)	0.63 (0.63–0.64)	0.7 (0.65–0.75)
Treatment	ADT	0.48 (0.47–0.49)	0.39 (0.35–0.42)	0.49 (0.48–0.50)	0.59 (0.53–0.65)	0.83 (0.83–0.84)	0.80 (0.76–0.83)	0.84 (0.83–0.84)	0.87 (0.82–0.91)
	DT	0.25 (0.23–0.28)	0.20 (0.13–0.29)	0.26 (0.23–0.28)	0.26 (0.11–0.45)	0.68 (0.65–0.71)	0.67 (0.55–0.77)	0.68 (0.65–0.71)	0.78 (0.54–0.90)
	RP	0.08 (0.06–0.09)	0.04 (0.02–0.07)	0.08 (0.07–0.10)	0.08 (0.01–0.23)	0.18 (0.16–0.19)	0.18 (0.13–0.23)	0.18 (0.16–0.19)	0.20 (0.07–0.37)
	RRT	0.11 (0.10–0.12)	0.04 (0.02–0.07)	0.12 (0.11–0.14)	0.15 (0.08–0.25)	0.31 (0.29–0.33)	0.28 (0.23–0.33)	0.31 (0.29–0.33)	0.36 (0.25–0.48)
Year of diagnosis	2000–2005	0.41 (0.39–0.42)	0.34 (0.28–0.40)	0.41 (0.39–0.42)	0.57 (0.49–0.65)	0.71 (0.70–0.72)	0.70 (0.64–0.76)	0.71 (0.69–0.72)	0.78 (0.70–0.84)
	2006–2014	0.33 (0.32–0.34)	0.19 (0.16–0.22)	0.34 (0.33–0.35)	0.38 (0.31–0.45)	0.62 (0.61–0.63)	0.49 (0.45–0.52)	0.63 (0.62–0.64)	0.68 (0.60–0.74)
	2015–2020	-	-	-	-	-	-	-	-

Radical prostatectomy (*RP*), radical radiotherapy (*RRT*), androgen deprivation therapy (*ADT*) or deferred treatment (*DT*). Cumulative risks with 95% confidence intervals

**Fig. 2:**
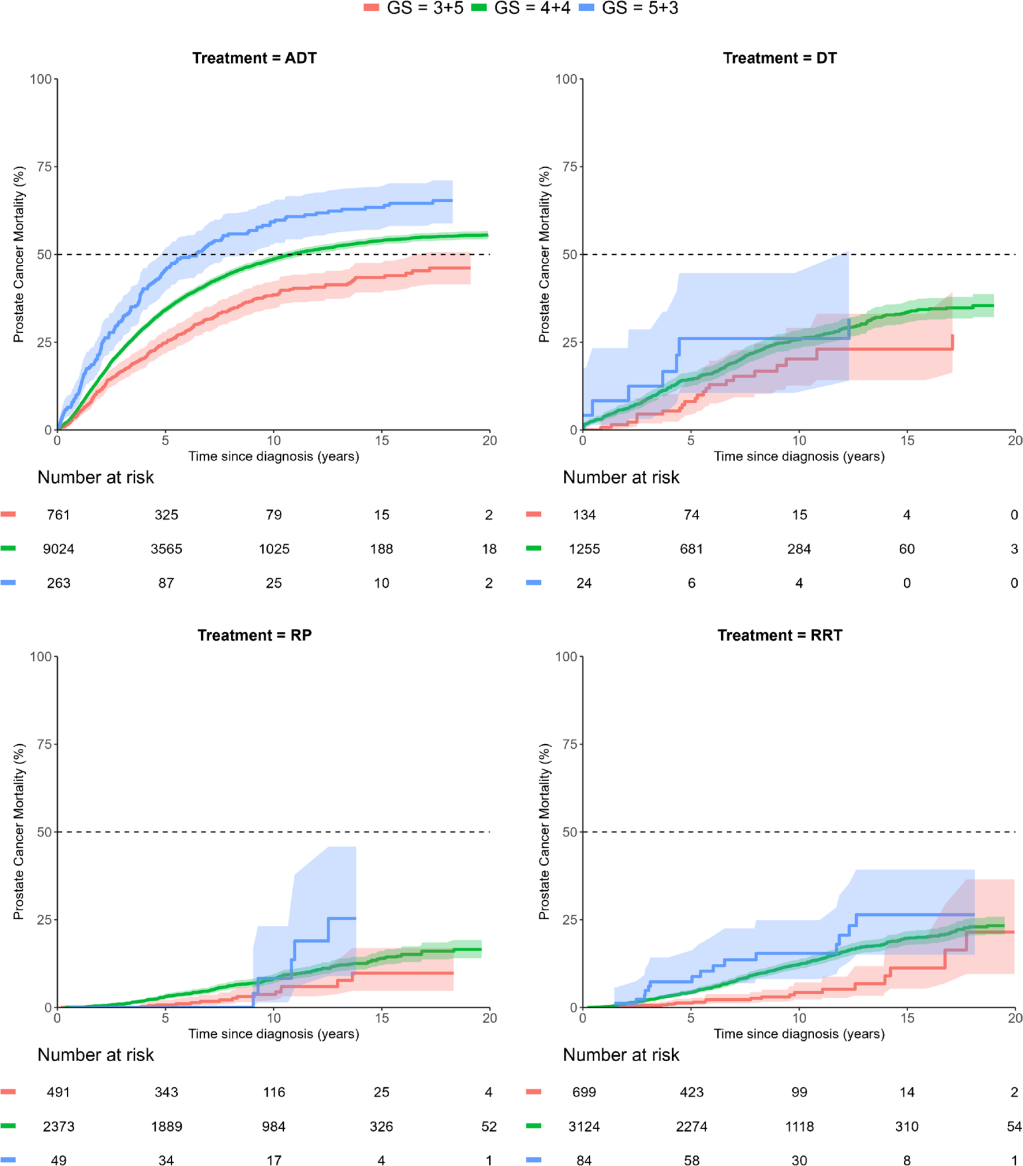
Prostate cancer-specific mortality (PCSM) of men with Gleason score 8 prostate cancer managed by androgen deprivation therapy (ADT), deferred therapy (DT), radical prostatectomy (RP) or radical radiotherapy (RRT), respectively. Curves are truncated at the time point of the last event

**Fig. 3:**
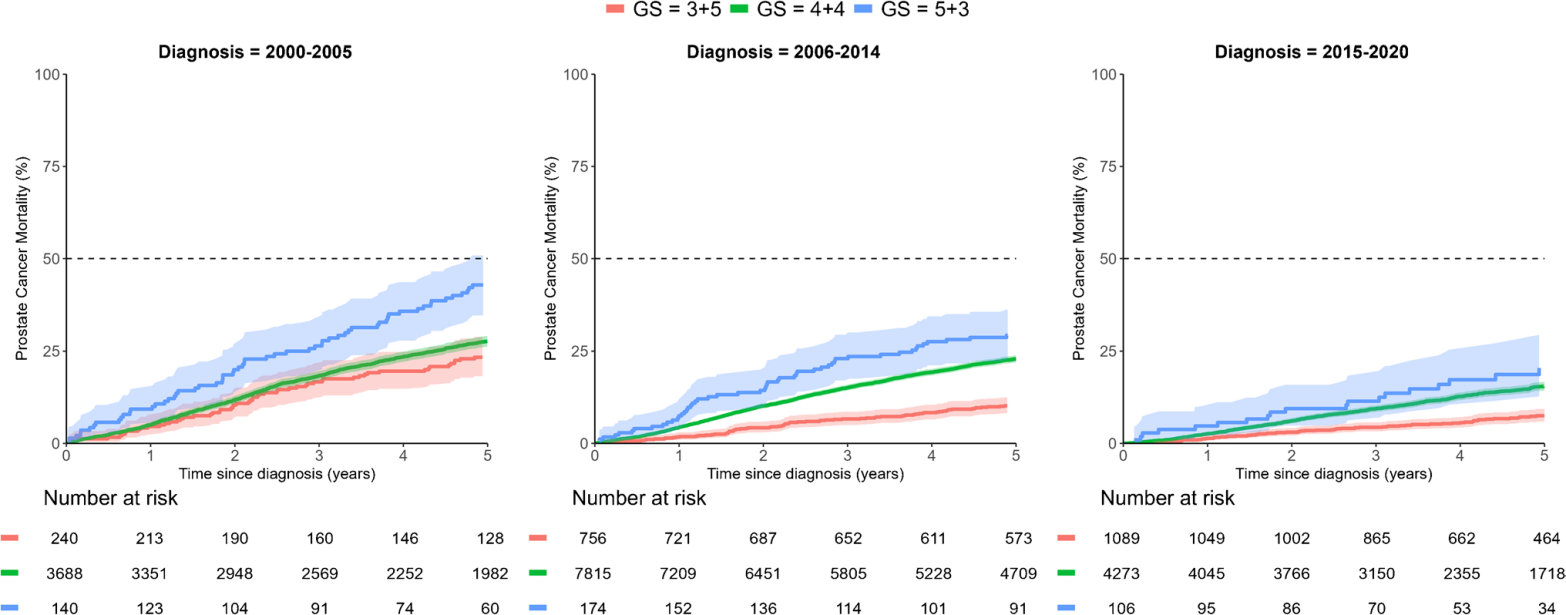
Prostate cancer-specific mortality (PCSM) of men with Gleason score 8 prostate cancer diagnosed in 2000–2005, 2006–2014 and 2015–2020, respectively. Curves are truncated at the time point of the last event

Separate analysis of constituent Gleason patterns showed substantial differences in PCSM. For men with a Gleason score of 5 + 3, the PCSM was higher than for those with a Gleason score of 4 + 4 after both 5- and 10-year follow-up. Similarly, the PCSM of men with a Gleason score of 4 + 4 was higher than for those with a Gleason score of 3 + 5. This pattern was repeated in men who had received ADT, while the mortality rate among men treated with radical prostatectomy or radical radiotherapy was generally so low that it was difficult to demonstrate significant differences in outcome (Table 2). In all men with Gleason score 8, cancers mortality was lower after radical prostatectomy or radical radiotherapy than after androgen deprivation therapy or deferred therapy (Table 2). Plots of PCSM and ACM after these treatment options are shown in Fig. 2 and Suppl. Figure 3, respectively. There was a substantial decrease in mortality from the first to the latest calendar period; plots of PCSM and ACM of all Gleason score 8 cancers diagnosed during the time periods of 2000–2005, 2006–2014 and 2015–2020, respectively, are shown in Fig. 3 and Suppl. Figure 4.

In a multivariable Cox analysis including Gleason score; age; cT, cN and cM staging categories; Charlson co-morbidity index; primary treatment; period of diagnosis; and education level, Gleason scores 4 + 4 and 5 + 3 are found to be significant predictors of PCSM, with hazard ratios (HR) of 1.32 (95% *CI* 1.18–1.49) and 1.96 (95% *CI* 1.62–2.37), respectively, utilising Gleason score 3 + 5 as the referent (Table 3).

**Table 3:** Multivariable Cox analysis of prostate cancer-specific (PCSM) and all-cause mortality (ACM) at 10 years

	PCSM	ACM
	HR (95% *CI*)	HR (95% *CI*)
Gleason score		
3 + 5	Ref	Ref
4 + 4	1.32 (1.18–1.49)	1.07 (0.99–1.16)
5 + 3	1.96 (1.62–2.37)	1.42 (1.23–1.63)
Age (per year increase)	1.02 (1.01–1.02)	1.04 (1.04–1.05)
T category		
T1c	Ref	Ref
T2	1.58 (1.42–1.76)	1.26 (1.18–1.35)
T3	2.12 (1.91–2.35)	1.50 (1.40–1.60)
T4	2.86 (2.54–3.23)	1.87 (1.72–2.04)
TX	2.19 (1.77–2.71)	1.49 (1.28–1.74)
N category		
N0	Ref	Ref
N1	1.58 (1.40–1.79)	1.31 (1.19–1.45)
NX	1.27 (1.15–1.40)	1.16 (1.08–1.24)
M category		
M0	Ref	Ref
M1	2.78 (2.59–2.98)	1.86 (1.76–1.97)
MX	1.20 (1.12–1.30)	1.15 (1.09–1.21)
CCI (per unit increase)	1.12 (1.10–1.14)	1.17 (1.16–1.18)
Treatment		
ADT	Ref	Ref
DT	0.67 (0.60–0.75)	0.85 (0.79–0.92)
RP	0.22 (0.18–0.26)	0.26 (0.24–0.30)
RRT	0.28 (0.25–0.32)	0.39 (0.36–0.42)
Year of diagnosis		
2000–2005	Ref	Ref
2006–2014	0.90 (0.85–0.96)	0.94 (0.90–0.98)
2015–2020	0.63 (0.57–0.69)	0.76 (0.71–0.82)
Education		
Low	Ref	Ref
Intermediate	0.91 (0.86–0.97)	0.93 (0.89–0.97)
High	0.90 (0.83–0.97)	0.87 (0.82–0.92)

Charlson co-morbidity index (*CCI*). Radical prostatectomy (*RP*), radical radiotherapy (*RRT*), androgen deprivation therapy (*ADT*) or deferred treatment (*DT*). Hazard ratios with 95% confidence intervals

## Discussion

In this population-based register study on the prognosis of Gleason score 8 prostate cancer in needle biopsies, there was a significantly higher mortality in men with Gleason score 5 + 3 cancer compared to those with 4 + 4 cancer and in men with Gleason score 4 + 4 cancer compared to those with 3 + 5 cancer. A majority of these men were treated with androgen deprivation therapy. Those who received treatment with curative intent in general had a very low prostate cancer mortality (0.04–0.15 after 10 years), and significant differences between the grade subgroups were not observed.

Prognostic heterogeneity in Gleason score 8 prostate cancer has been demonstrated in most previous studies [2–7]; however, outcomes between Gleason scores 3 + 5, 4 + 4 and 5 + 3 vary between the studies. In a systematic review and meta-analysis, Lu et al. analysed eight reports and concluded that the meta-analysis was hampered by the widely differing methodologies of the studies [4]. In particular, there were differences in the specimens used for the studies with cases consisting of biopsy specimens [2, 3, 5, 7, 8], radical prostatectomy specimens [2] or a combination of the two [6]. Other differences were that some studies relied on single-centre data [3], while others were based on multiple-centre data [2, 8] or registry data [5–7]. A problem in several studies was the low number of some of the grade categories. This was in particular true for Gleason score 5 + 3 tumours, and as a consequence, results from these studies need to be interpreted with caution [5, 8]. Selection bias most likely accounts for some observed outcome differences between treatments, such as the low prostate cancer mortality in men who underwent radical prostatectomy or radical radiotherapy in the current study.

Using Surveillance, Epidemiology and End Results (SEER) database registry data, Mahal et al. found that Gleason score 5 + 3 cancers had a significantly worse prognosis than those with a Gleason score of 3 + 5 or 4 + 4 [6]. However, the methodology of grade reporting in SEER was not uniform throughout the study period, and grading was based on the highest Gleason score in either the preoperative needle biopsies or the subsequent radical prostatectomy specimen. As a consequence, the results of this latter study are not comparable with those of our study. By contrast, Gandaglia et al. found a similar outcome in tumours with Gleason scores 4 + 4 and 5 + 3, but their data were based on radical prostatectomy specimens only [2]. Huynh et al. and Rusthoven et al. on the other hand combined cases of Gleason scores 3 + 5 and 5 + 3 and compared them against outcomes of Gleason score 4 + 4 tumours based on the assumption that the presence of a Gleason pattern 5 would be the most critical marker of prognosis [3, 7].

Gleason score 8 cancers on needle biopsy are Gleason score 4 + 4 in the vast majority of cases, and only 9% [3]–21% [8] are being reported as 3 + 5 or 5 + 3. In the present study, 86% were 4 + 4, which is in line with the other large register-based study [6]. Of all Gleason score 8 cancers in earlier series, only ≤ 5% were reported as 5 + 3 [5, 6, 8]. As noted above, some of the previous studies merged Gleason scores 3 + 5 and 5 + 3 due to a paucity of cases and did not report separate results [3, 7].

At the 2014 ISUP consensus conference on Gleason grading, it was recommended that the grading of prostate cancer be based on 5 ISUP grades (also known as grade groups) for reporting purposes. These so-called ISUP grade groups were defined on the basis of Gleason scores, i.e. Grade 1, Gleason score 3 + 3 = 6; Grade 2, Gleason score 3 + 4 = 7; Grade 3, Gleason score 4 + 3 = 7; Grade 4, Gleason scores 3 + 5 = 8, 4 + 4 = 8 or 5 + 3 = 8; and Grade 5 4 + 5 = 9, 5 + 4 = 9 or 5 + 5 = 10. It is apparent that while ISUP grades 1–3 consist of single Gleason scores, this does not apply to ISUP grades 4 and 5, and concerns have been raised that this grouping would have considerable disadvantages by not revealing the exact Gleason grade composition present in individual cases [16]. It has been shown that any percentage of Gleason pattern 5 tumour in a needle biopsy is associated with a worse prognosis when compared to Gleason score 4 + 3 tumours [17]. In a recent study, we analysed the outcome of ISUP grade 5 cancers and found significant differences in mortality between the three grade subgoups (Gleason scores 4 + 5, 5 + 4 and 5 + 5) confirming that a grouping of these Gleason scores would cause loss of granularity of data which, in turn, would diminish any prognostic information derived from grading [12]. In this present study, we found further support for this hypothesis. By restricting our analysis to the subgroups of tumours that constitute ISUP grade 4, we now also demonstrated a prognostic heterogeneity within this ISUP grade. Interestingly, the PCSM for Gleason score 5 + 3 cancers was very similar to that of Gleason score 4 + 5 cancers, reported in our earlier study, with PCSM after 10 years at 0.44 (0.39–0.49) and 0.45 (0.44–0.46), respectively [12].

Thus, while we have demonstrated significant differences in prognosis between the components of ISUP grades 4 and 5 when analysed separately, there was also an overlap between some components of ISUP grade 4 and ISUP grade 5. These data further emphasise that by grouping of Gleason scores to generate ISUP grades, valuable prognostic information is lost.

### Supplementary Information

Below is the link to the electronic supplementary material.

Supplementary file1 (PPTX 39 KB)

Supplementary file2 (PNG 176 KB)

Supplementary file3 (PNG 618 KB)

Supplementary file4 (PNG 350 KB)
