# Supplementary material for: Prognosis of Gleason score 8 prostatic adenocarcinoma in needle biopsies: a nationwide population-based study
Source: Virchows Arch. 2024 Apr 29;484(6):995–1003. doi: 10.1007/s00428-024-03810-y (PMC11186860; doi:10.1007/s00428-024-03810-y)
Supplement: Supplementary file 1 — Supplementary file1 (PPTX 39 KB) [file 428_2024_3810_MOESM1_ESM.pptx]

## Slide 1
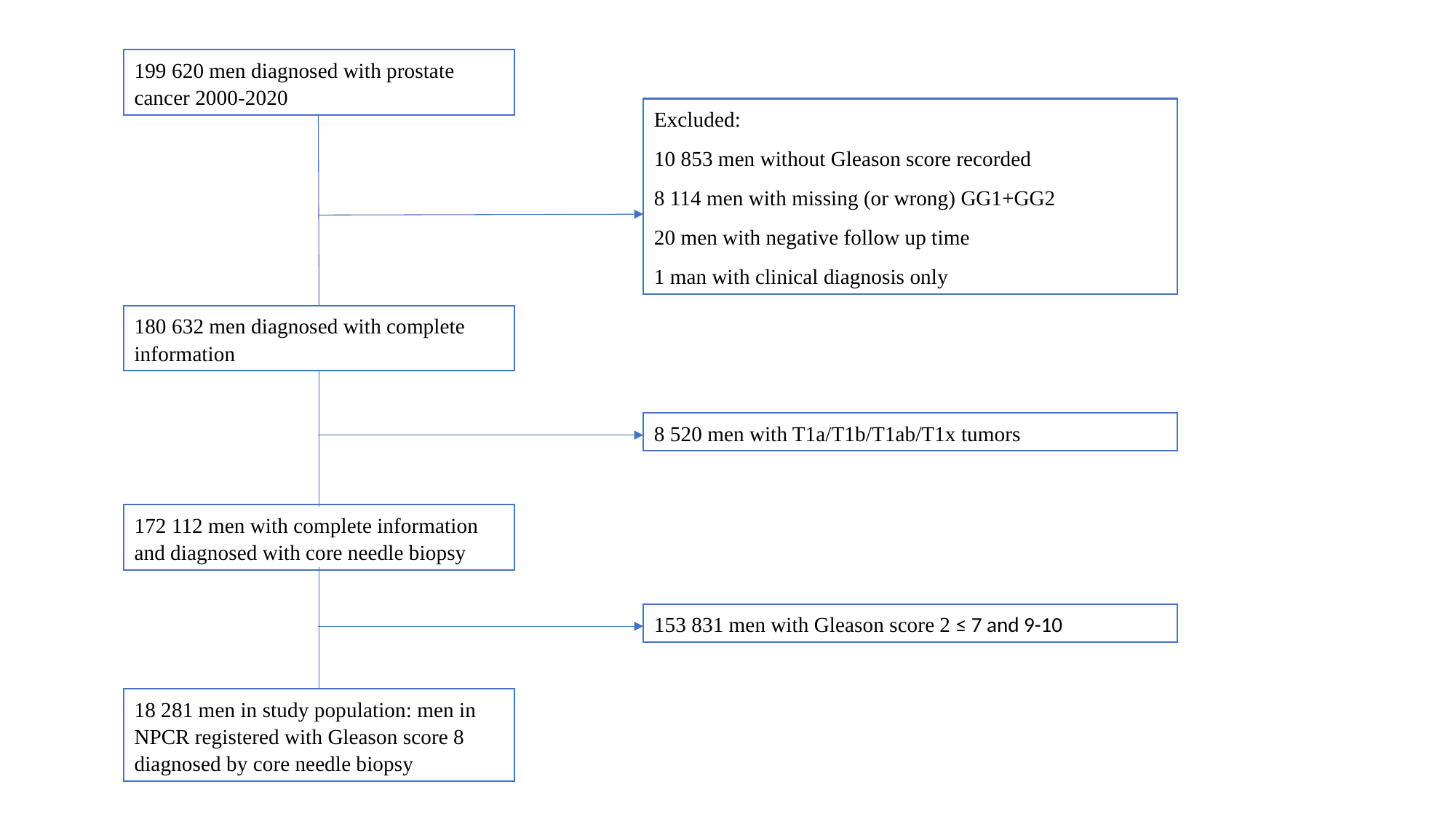

199 620 men diagnosed with prostate cancer 2000-2020
Excluded:
10 853 men without Gleason score recorded
8 114 men with missing (or wrong) GG1+GG2
20 men with negative follow up time
1 man with clinical diagnosis only
180 632 men diagnosed with complete information
8 520 men with T1a/T1b/T1ab/T1x tumors
172 112 men with complete information and diagnosed with core needle biopsy
153 831 men with Gleason score 2 ≤ 7 and 9-10
18 281 men in study population: men in NPCR registered with Gleason score 8 diagnosed by core needle biopsy
