# Supplementary figures and images for: Prognosis of Gleason score 8 prostatic adenocarcinoma in needle biopsies: a nationwide population-based study
Source: Virchows Arch. 2024 Apr 29;484(6):995–1003. doi: 10.1007/s00428-024-03810-y (PMC11186860; doi:10.1007/s00428-024-03810-y)

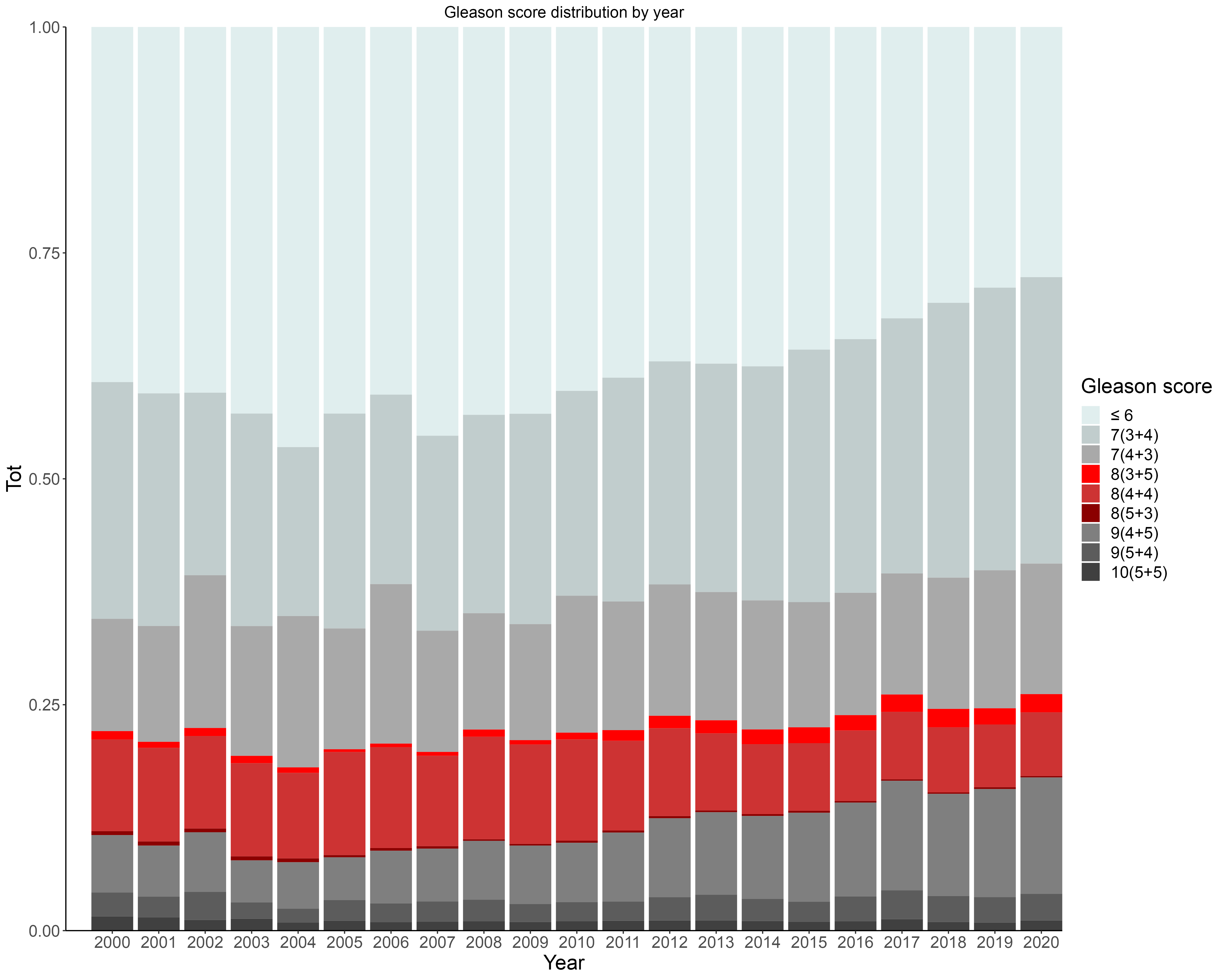

Supplement: Supplementary file 2 — Supplementary file2 (PNG 176 KB) [file 428_2024_3810_MOESM2_ESM.png]

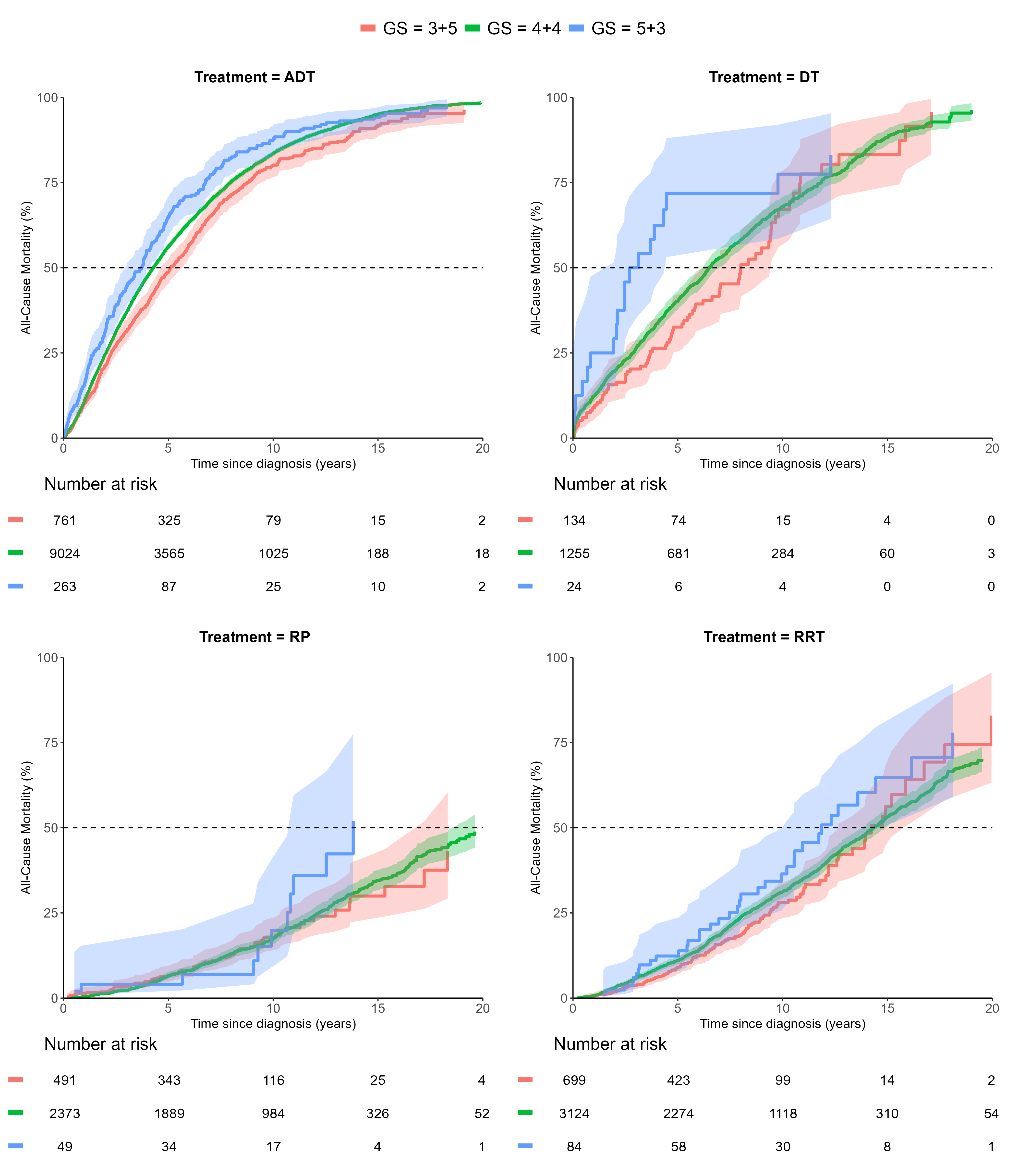

Supplement: Supplementary file 3 — Supplementary file3 (PNG 618 KB) [file 428_2024_3810_MOESM3_ESM.png]

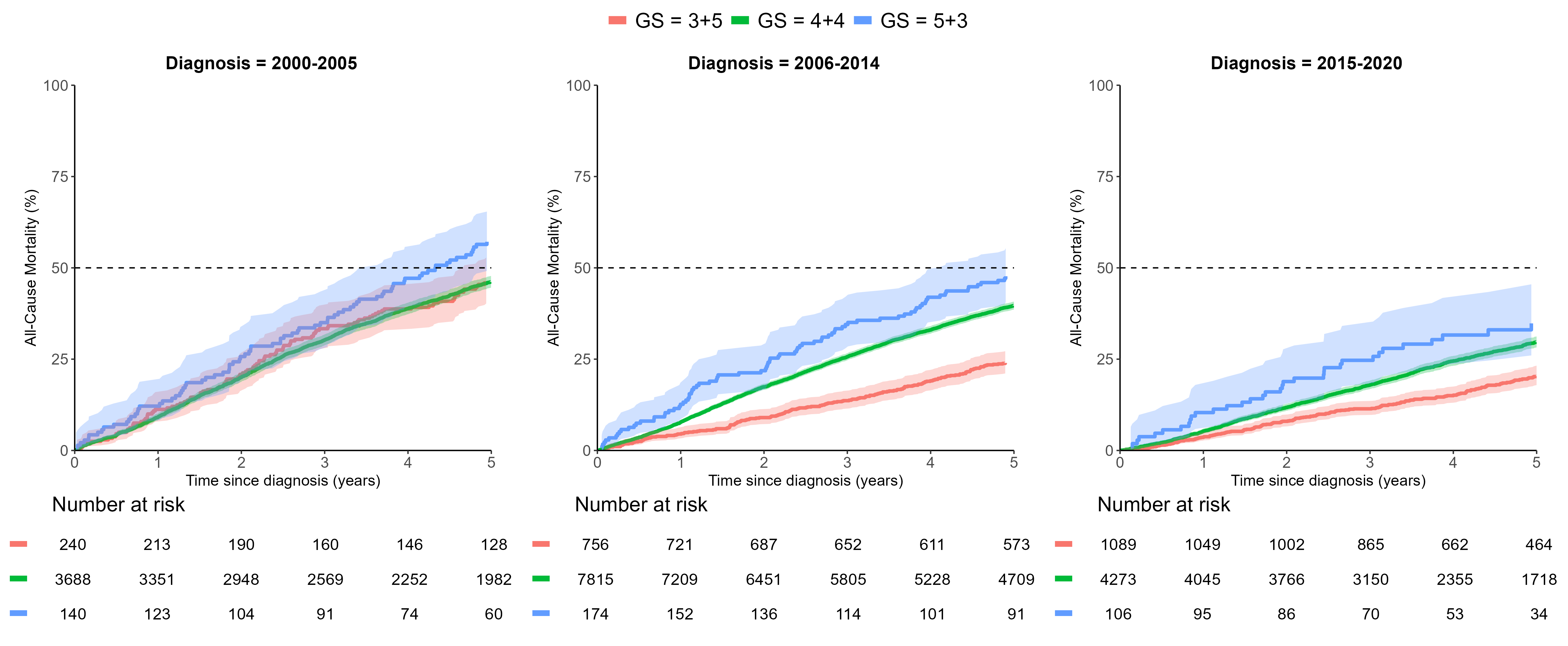

Supplement: Supplementary file 4 — Supplementary file4 (PNG 350 KB) [file 428_2024_3810_MOESM4_ESM.png]
